# L-FABP as a Potential Biomolecular Marker of Liver Graft Injury

**DOI:** 10.3390/jcm14207404

**Published:** 2025-10-20

**Authors:** Ana Kalamutova, Danaja Plevel, Mihajlo Djokic, Ales Jerin, Blaž Trotovšek, Miha Petric

**Affiliations:** 1 Department of Abdominal Surgery, University Medical Centre Ljubljana, Zaloska 7, 1000 Ljubljana, Slovenia; ana.kalamutova@gmail.com (A.K.); danaja.plevel@kclj.si (D.P.); mihajlo.djokic@kclj.si (M.D.); blaz.trotovsek@kclj.si (B.T.); 2 Faculty of Medicine, University of Ljubljana, Vrazov Trg 2, 1000 Ljubljana, Slovenia; 3 Institute of Clinical Chemistry and Biochemistry, University Medical Centre Ljubljana, 1000 Ljubljana, Slovenia; ales.jerin@kclj.si; 4 Faculty of Pharmacy, University of Ljubljana, 1000 Ljubljana, Slovenia

**Keywords:** liver transplantation, biomarker, L-FABP, hepatic graft injury, marginal liver graft

## Abstract

**Background**: In recent years, indications for liver transplantation have expanded, while the age of transplant recipients has significantly increased due to improvements in perioperative management. As clinical manifestations of posttransplant complications vary and are often nonspecific, the identification of appropriate biomarkers is important for the assessment of graft quality and early recognition of potential complications following liver transplantation. Liver-type FABP (L-FABP) is a small cytoplasmic protein found abundantly in hepatocytes and is involved in the intracellular transport of long-chain fatty acids. Elevated serum levels have been detected in acute and chronic liver failure, kidney failure, and some malignancies. **Materials and Methods**: We conducted a prospective, single-center study from July 2023 to January 2025, including 29 adult patients who underwent deceased-donor transplantation. Three patients were excluded due to inadequate sample withdrawals. Serum L-FABP was measured preoperatively and on postoperative days 1, 3, 5, 7, and 14. Clinical, surgical, and biochemical data were collected and analyzed using non-parametric statistical tests. **Results**: L-FABP levels were significantly higher on POD 7 in recipients of grafts from donors ≥ 65 years (*p* = 0.035), with no corresponding changes in standard liver function markers. While no significant differences in L-FABP levels were found between patients with and without infectious biliary or vascular complications (all *p* > 0.05), we proved a strong negative correlation between intraoperative blood transfusion volume and L-FABP levels on POD 5 (ρ = −0.677, *p* < 0.001) and POD 7 (ρ = −0.455, *p* = 0.025). **Conclusions**: Our findings suggest that L-FABP holds promise as a biomarker for the early detection of subclinical hepatic graft cellular injury, which is not detected by means of conventional biomarkers for liver function.

## 1. Introduction

Since 1963, when Starzl first performed the procedure, liver transplantation has remained the only curative treatment for patients with chronic liver disease, end-stage liver disease, or acute liver failure [1,2,3]. It has gained acceptance in liver transplant oncology, showing promising outcomes in treating hepatocellular carcinoma and, in recent decades, in managing inoperable colorectal and neuroendocrine liver metastases, as well as intrahepatic and perihilar cholangiocarcinoma [2,4,5]. The growing demand for liver transplantation, driven by a wide range of medical indications, has resulted in a significant shortage of available organs for transplantation. To address this issue, several strategies have been proposed to expand the liver graft pool, including split liver transplantation, living liver donation, donation after circulatory death, dynamic machine preservation, and the use of older donors [6,7,8,9,10]. The global increase in recipient and donor age has led to several retrospective analyses exploring the postoperative outcomes of using older grafts, indicating higher rates of early mortality in older grafts and greater graft-recipient age mismatch [11,12,13]. Recent research has focused on the cellular quality of marginal liver grafts, which remains a significant concern [10]. The clinical manifestations of post-transplant complications vary and are nonspecific. Ischemic cholangiopathy can have a non-specific or even asymptomatic initial presentation, appearing only in abnormal liver function tests [14]. Conventional biochemical markers for biliary complications include elevated levels of bilirubin, alkaline phosphatase (AP), alanine transferase (ALT), aspartate transferase (AST), gamma-glutamyl transferase (GGT), and other inflammatory markers [15]. Among the imaging modalities, Magnetic Retrograde Cholangiopancreatography (MRCP) is considered the gold standard for diagnosing biliary complications [15,16]. Computed tomography (CT) is used to diagnose infectious and vascular complications [17]. The identification of suitable prognostic biomarkers is essential for assessing liver graft quality and enabling the early detection of potential complications following liver transplantation [18]. Evaluating cellular-level function is vital for diagnosing the causes of graft injury, guiding immunosuppression, and improving overall patient survival rates [18]. Biomarkers can be detected in the extracellular environment of various biological fluids, such as bile, blood, urine, and perfusate, allowing for straightforward detection and eliminating the need for liver graft biopsies, thus reducing potential postbiopsy complications [19]. Fatty acid binding proteins (FABP) are small cytoplasmic proteins primarily responsible for facilitating the intracellular transport of long-chain fatty acids and are abundantly expressed in tissues with active fatty acid metabolism. To date, nine isoforms have been identified and named according to the organ in which they were initially discovered, although some have been found in multiple organs [20]. While the intestinal type (I-FABP) is found only in the intestine, intestinal enterocytes exhibit a higher abundance of liver-type FABP (L-FABP), which was originally identified in the liver and has since been discovered in renal tubular cells and predominantly in duodenal and jejunal enterocytes [20,21]. Through interactions with peroxisome proliferator-activated receptors (PPARs), L-FABP mediates inflammation [22]. Its serum levels have been found to be elevated in cases of colon, liver, gastric, lung, and breast cancer [23,24]. Serum and, in some cases, urinary L-FABP has been investigated as a potential biomarker for acute and chronic kidney failure, necrotizing enterocolitis, abdominal trauma, drug-induced liver failure, and acute chronic liver failure [25,26,27,28,29,30,31,32,33]. FABPs have emerged as potential biomarkers for tissue injury because of their predominant cytoplasmic localization, low serum concentration, and organ specificity [20]. A study on porcine liver transplants revealed that L-FABP is associated with warm ischemia and hepatocellular damage within the first 180 min after reperfusion [34]. The heart isoform, H-FABP, was the first to show elevated serum levels in myocardial injury and has since been recognized as a potential biomarker for acute myocarditis, heart failure, heart injury in polytrauma, decreased kidney function in cardiac patients, and transient ischemic attack [35,36,37,38,39,40]. Additionally, FABPs are intriguing potential biomarkers because studies have demonstrated that their serum levels rise earlier than those of other conventional biomarkers, potentially leading to earlier detection of injury or disease [35,38]. Recent research has underscored the utility of FABP as a small-molecule biomarker that appears in plasma sooner than traditional biomarkers, thus facilitating the early detection of cellular injury. Our research aimed to investigate serum FABP levels over an extended perioperative period and analyze their temporal trends in comparison with conventional biomarkers. We designed a study to explore the relationship between serum L-FABP levels and complications following liver transplantation, aiming to determine whether FABP could serve as a potential biomarker for hepatic graft injury. The secondary aim of our study was to identify the correlation between serum L-FABP and conventional biochemical parameters to assess its utility as a diagnostic tool for hepatic graft injury following liver transplantation.

## 2. Materials and Methods

### 2.1. Study Design and Protocol

**Figure 1:**
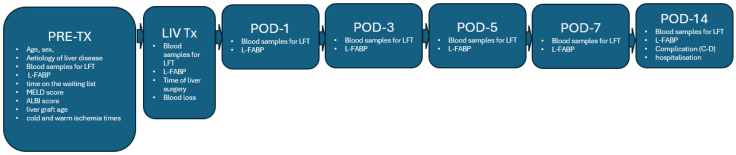
Flowchart of study data collection.

We conducted a prospective, single-center study from July 2023 to January 2025 at the Department of Abdominal Surgery, University Medical Center, Ljubljana. The study included 29 consecutive patients aged ≥ 18 years who underwent liver transplantation for various reasons. All participants received whole-liver grafts from donors after brain death (DBD). A prospective database was created according to the study protocol. After obtaining informed consent, we collected preoperative parameters, including recipient age, sex, liver disease etiology, time on the waiting list, MELD score [41], and ALBI score [42]. We also recorded graft age, duration of surgery, cold and warm ischemia times, and need for blood transfusions during the procedure. Laboratory parameters were evaluated at admission and on postoperative days (POD) 1, 3, 5, 7, and 14 following liver transplantation (LT), including lactate, total and direct bilirubin, ALP, GGT, ALT, AST, C-reactive protein (CRP), and leukocyte count (Figure 1). 

All patients were treated according to the institutional LT protocol. Routine Doppler ultrasound (US) was performed on POD 1 unless computed tomography (CT) was necessary due to suspected early complications. Following LT, we documented the duration of stay in the intensive care unit (ICU) and hospital, postoperative complications categorized using the Clavien–Dindo classification [43], infections, and 90-day mortality, all of which were extracted from the liver recipients’ database for analysis. The diagnosis of liver rejection was confirmed through laboratory tests and histological examination of liver graft biopsy specimens obtained via ultrasound-guided biopsy. Acute rejection was defined as a score of ≥6 points according to the liver allograft fibrosis score [44].

### 2.2. Sample Processing

Venous blood samples for serum FABP analysis were obtained on the day of surgery upon admission to the hospital ward and on postoperative days (POD) 1, 3, 5, 7, and 14. Blood samples were collected without an additive. Serum was separated after centrifugation (1500× *g* for 10 min) and aliquots were stored at −20 °C until analyses. The concentrations of L-FABP were measured using ELISA (CusaBio Technology Ltd., Houston, TX, USA) with the detection limit of 7.8 ng/L; CVs within series were <10%.

### 2.3. Statistical Analysis

The data were analyzed using IBM SPSS Statistics version 29.0 (IBM Corp., Armonk, NY, USA) and Jamovi version 2.4.5 (The Jamovi Project, Sydney, Australia), an open-source statistical platform based on R. The normality of the continuous variables was assessed using the Shapiro–Wilk test, which is recommended for small samples (*n* < 50). The results showed that most variables were not normally distributed (*p* < 0.05), which is why non-parametric tests were used throughout. The Mann–Whitney U test was used to compare FABP isoform values and MELD/ALBI scores between independent groups. Spearman’s rank correlation coefficient was used to assess associations between FABP values and continuous variables such as age, MELD/ALBI score, surgical time, and laboratory parameters. The chi-square test was used to assess associations between categorical variables (e.g., complication rates by donor age or etiology). In selected cases where multiple independent variables were included, General Linear Models were used to examine the effects and possible interactions with gender. Effect sizes were reported using partial eta squared (η 2 p) for General Linear Models and r for Mann–Whitney U tests. A significance level of *p* < 0.05 was used for all statistical tests.

### 2.4. Ethical Statement

This study was approved by the National Ethics Committee (0120-493/2021/3) and conducted in accordance with the Declaration of Helsinki. Prior to participation, all OF the patients provided verbal and written informed consent.

## 3. Results

### 3.1. Patient Demographics

The study sample comprised 29 participants, of whom 62.1% were male (n = 18) and 37.9% were female (n = 11). The mean age of the participants was 55.21 years (SD = 11.88), with an age range of 23–70 years. On average, 383.31 days (SD = 155.34) had elapsed since the participants underwent liver transplantation, with a range of 89–630 days. The mean waiting period prior to transplantation was 115.97 days (SD = 154.60), with a range of 0–630 days. Three patients were excluded from the FABP analysis because of incomplete blood sample data. Notably, four of the included cases involved re-transplantation. The age of the donors ranged from 14 to 87 years, with a mean age of 60.14 years (SD = 17.42). Baseline patient characteristics and intraoperative details of liver transplant recipients are shown in Table 1.

### 3.2. Preoperative Values of L-FABP

Spearman’s correlation analysis indicates that there is no statistically significant association between preoperative FABP-L levels and recipient age (ρ = −0.110, *p* = 0.591). However, a moderate negative correlation was observed between preoperative FABP-L levels and MELD score (ρ = −0.344), although this result did not reach statistical significance (*p* = 0.085) (Table 2).

We identified a statistically significant moderate negative correlation between preoperative FABP-L levels and the ALBI score (ρ = −0.397, *p* = 0.045) (Table 3).

The Mann–Whitney U test indicated no statistically significant difference in preoperative FABP-L levels between patients with alcoholic and non-alcoholic liver failure (U = 63.0, *p* = 0.391, r = 0.21). Similarly, no significant difference was observed between patients with and without major postoperative complications (Clavien–Dindo grade ≥ 3) during hospitalization (U = 69.0, *p* = 0.462, r = 0.17) or between patients with and without malignant liver or bile duct disease (U = 66.0, *p* = 1.000, r = 0.00).

### 3.3. Graft Age

The Mann–Whitney U test indicated no statistically significant differences in FABP-L levels post-surgery between grafts from donors younger than 65 and those aged 65 and older at most postoperative time points (POD 1, 3, 5, and 14; all *p* > 0.05). A statistically significant difference was identified on POD 7 (U = 32.0, *p* = 0.035, r = 0.52), with elevated FABP-L levels observed in the group aged 65 and older (Table 4).

**Figure 2:**
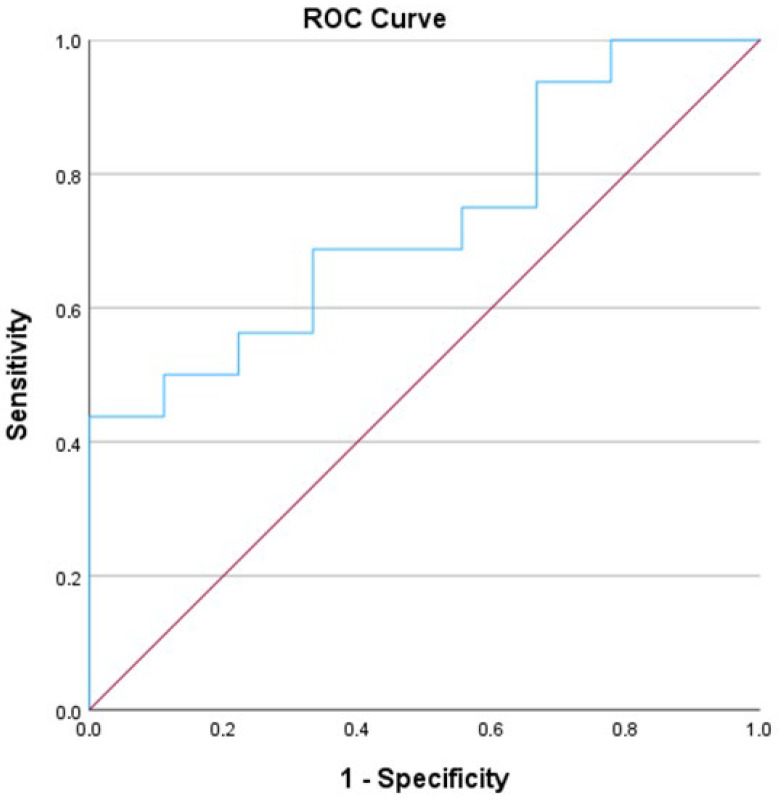
ROC curve for L-FABP on POD 7 in graft age over 65. The area under the curve is 0.729.

Additionally, we conducted a Receiver Operating Characteristic (ROC) analysis, which revealed an Area Under the Curve (AUC) of 0.729 (Figure 2).

**Figure 3:**
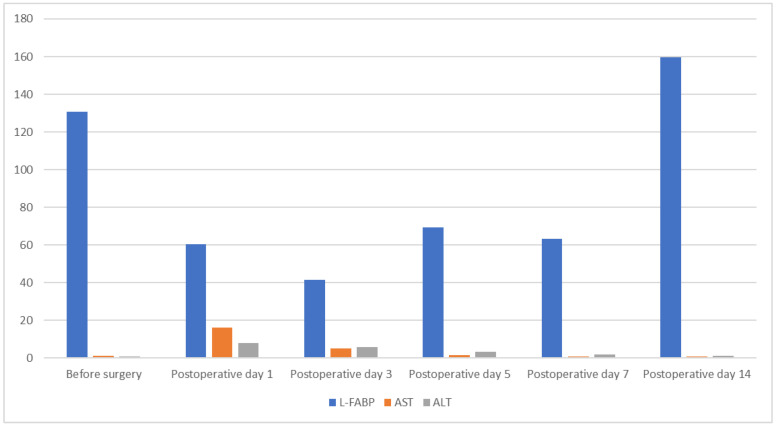
Graph depicting trends of L-FABP compared to transaminases. L-FABP. Liver-type fatty acid binding protein; AST—aspartate aminotransferase; ALT—alanine aminotransferase.

In light of the statistically significant difference in FABP-L levels observed on POD 7 between grafts from donors aged below and above 65 years (U = 32.0, *p* = 0.035, r = 0.52), conventional clinical biochemical markers were also evaluated at the same time point to determine if a similar trend could be identified (Figure 3).

However, no statistically significant differences were detected between the age groups for any of the markers, including AST, ALT, leukocyte count, CRP, total and direct bilirubin, GGT, and alkaline phosphatase (all *p* > 0.05).

### 3.4. Duration of Surgery, Cold and Warm Ischaemia Duration

Spearman’s correlation coefficients revealed no statistically significant associations between FABP-L levels at any postoperative time point (POD 1, 3, 5, 7, and 14) and the duration of cold ischemia, warm ischemia, and total surgery time (all *p*-values exceeding 0.05).

### 3.5. Intraoperative Blood Loss

Spearman’s correlation revealed significant negative associations between intraoperative blood transfusion volume and FABP-L levels on postoperative days 5 and 7. Specifically, FABP-L levels on POD 5 showed a strong negative correlation with transfused blood volume (ρ = −0.677, *p* < 0.001), while POD 7 showed a moderate negative correlation (ρ = −0.455, *p* = 0.025) (Table 5).

**Figure 4:**
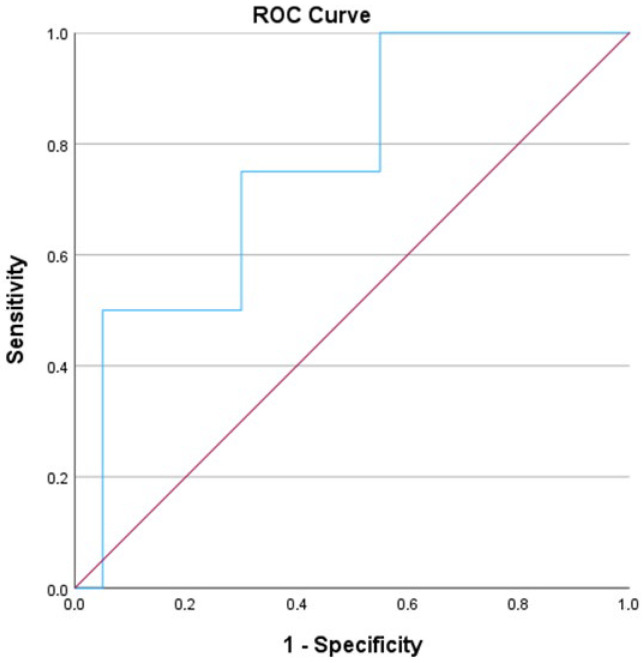
ROC curve for L-FABP on POD 1 and postoperative hemorrhage. Area under the curve is 0.763.

No significant correlations were identified on POD 1, 3, or 14 (all *p* > 0.05). These findings suggest that increased intraoperative blood loss may be associated with reduced FABP-L levels at specific postoperative intervals, such as POD 5 and POD 7. Despite the significant negative correlation between FABP-L levels on POD 5 and the volume of intraoperative blood transfusion (ρ = −0.677, *p* < 0.001), none of the conventional biochemical markers assessed on the same postoperative day exhibited statistically significant associations with blood loss (all *p* > 0.05). Spearman’s correlations for AST, ALT, CRP, leukocyte count, total and direct bilirubin, GGT, and alkaline phosphatase on POD 5 ranged from −0.322 to 0.316, yet none achieved statistical significance. Additionally, we conducted a Receiver Operating Characteristic (ROC) analysis, which revealed an Area Under the Curve (AUC) of 0.763 (Figure 4).

Following the observed moderate negative correlation between intraoperative blood transfusion volume and FABP-L levels on postoperative day 7 (ρ = −0.455, *p* = 0.025), additional analyses were performed to investigate associations between FABP-L on POD 7 and selected conventional biochemical and inflammatory markers. Among these, only the leukocyte count demonstrated a statistically significant correlation with FABP-L levels (ρ = 0.508, *p* = 0.011), while no significant associations were found for AST, ALT, CRP, total and direct bilirubin, GGT, or alkaline phosphatase (all *p* > 0.05).

### 3.6. Postoperative Complications

Postoperatively, we recorded Clavien–Dindo grade 3–4 complications in 14 patients (53.8%). In 11 (42.3%) patients, we proved an intra-abdominal abscess. Biliary complications, including biliary anastomosis stenosis or dehiscence, biliary leak, and cholangitis, were observed in eight patients (30.8%), and liver vascular complications were observed in four patients (15.4%). We recorded postoperative hemorrhage, which required intervention in five patients (19.2%). Graft rejection was proven with biopsy in one patient (3.8%). Four patients (15.4%) became candidates for re-transplantation. The 30-day readmission and morbidity rate was 0.0%.

The Mann–Whitney U tests for FABP-L indicated no significant differences between patients with and without complications during their hospital stay across all time points, with *p*-values ranging from 0.462–0.742. The effect sizes, ranging from 0.07 to 0.18, suggest minimal and clinically insignificant intergroup differences. Furthermore, the Mann–Whitney U test revealed no statistically significant differences in FABP-L levels between patients who developed post-surgery biliary complications and those who did not (all *p* > 0.05). Similarly, the Mann–Whitney U test showed no statistically significant differences in FABP-L levels between patients with and without postoperative bleeding at any of the measured time points (POD 1, 3, 5, 7, and 14; all *p* > 0.05). Additionally, the Mann–Whitney U test did not reveal any statistically significant differences in FABP-L levels between patients with and without vascular complications at any postoperative time point (POD 1, 3, 5, 7, and 14; all *p* > 0.05). The Mann–Whitney U tests comparing FABP-L levels between patients with and without confirmed graft rejection showed no significant differences on any of the measured postoperative days (POD), with *p*-values ranging from 0.077 to 0.462. Similarly, the Mann–Whitney U tests comparing FABP-L levels between patients with and without suspected graft rejection showed no significant differences on any of the measured postoperative days (POD), with *p*-values ranging from 0.157 to 0.801. Lastly, the Mann–Whitney U tests comparing FABP-L levels between patients with and without intra-abdominal abscesses following surgery showed no statistically significant differences at any postoperative day (POD), with *p*-values ranging from 0.259 to 0.919. Table 6 presents a summary of the key findings and statistical significance concerning postoperative complications.

## 4. Discussion

To the best of our knowledge, this is the first study to evaluate the clinical utility of L-FABP as a biomarker for the perioperative management of patients following LT. The primary aim of this study was to assess the potential of serum L-FABP as an indicator of hepatic graft cellular injury after LT. A notable limitation of our study is the small sample size, which restricts our ability to achieve statistically significant results. Nevertheless, our research indicates that even without definitive statistical significance, L-FABP demonstrates increased sensitivity in specific clinical contexts, such as graft age and blood loss, compared to conventional blood tests, and may serve as a potential marker of hepatocyte injury.

The implementation and expansion of LT indications have led to a shortage of available grafts [3]. To address this issue, various strategies have been employed to increase the availability of potential liver grafts [45]. Recent studies have primarily focused on evaluating the quality of liver grafts. Several methods are currently available for assessing the functional status of liver grafts [46].

Commonly used liver function tests include ALT, AST, ALP, GGT, serum bilirubin, prothrombin time (PT), international normalized ratio (INR), total protein, and albumin levels [47]. ALT and AST are primarily found in the liver; however, ALT is also present in lower concentrations in cardiac, renal, and muscle tissues, making it more specific to hepatocellular injury [48]. In contrast, AST is not only found in the liver but also in higher concentrations than ALT in the skeletal muscle, cardiac muscle, renal tissue, and brain [49]. Gamma-glutamyl transferase (GGT) is an enzyme present in various organs, including the pancreas, seminal vesicles, kidneys, biliary tract, and liver. Elevated serum concentrations and increased AST and ALT levels suggest hepatobiliary disease [50]. Using these three markers, we can accurately determine whether liver injury follows a hepatocellular or cholestatic pattern [51,52]. However, due to their presence in other tissues or organs, these markers are not specific and can be underestimated or falsely elevated in other clinical scenarios such as bleeding, myocardial infarction, or muscle injury. The accurate static indicators of liver function are serum albumin, bilirubin, and INR levels [53,54,55]. These markers reflect synthetic liver function; however, they can be influenced by various extrahepatic factors, such as malnutrition, extrahepatic bile duct obstruction, hemolysis, and the effects of anticoagulant therapy. Currently, liver biopsy is the only objective method for assessing the quality of the liver parenchyma and identifying potential signs of acute or chronic rejection, necrosis, and biliary complications [56,57,58]. An experienced pathologist is essential for accurately analyzing liver biopsy samples. The Banff scoring system is the most commonly used system for determining the grade of liver rejection [59]. However, the procedure is invasive and associated with several potential complications, including post-procedure bleeding, biliary leakage, and perforation of a hollow organ [19].

The existing literature offers limited evidence of a negative correlation between L-FABP and blood loss, as observed in the present study cohort (Table 5). In research involving preterm neonates with anemia, higher L-FABP levels were associated with lower hemoglobin levels [60]. Further studies have suggested a positive correlation between L-FABP and hemorrhagic shock in cases of abdominal trauma, as well as in isolated kidney and liver injuries [32]. Another study evaluated the use of I-FABP and L-FABP in emergency trauma settings for detecting abdominal trauma and noted increased serum FABP levels upon admission [33]. In both studies, serum FABP levels were measured only up to the second post-traumatic day [32,33]. Unlike previous studies, our study found a statistically significant reduction in serum FABP levels on postoperative days (POD) 5 and 7 in patients who received transfusions. However, the literature does not offer a satisfactory explanation for this finding. One possible explanation is that a higher volume of transfusion may cause a washout effect, reducing the detectable plasma levels of the FABP-L isomer. Although this observation seems contradictory, as one might expect major transfusion to be associated with hemodynamic instability and subsequent tissue hypoperfusion, leading to elevated plasma levels of FABP-L, it remains promising because no other conventional markers showed significant changes. An alternative explanation could be a statistical error, potentially resulting in inaccurate results due to the small sample size. This finding requires validation but suggests a possible superior sensitivity to cellular-level changes compared with conventional markers of liver function.

Marginal liver grafts are more susceptible to ischemia/reperfusion injury (IRI), leading to an early decline in hepatic cells. This process begins with hypoxia-induced metabolic dysfunction and is aggravated by hyperdynamic stress during reperfusion. The identification of early allograft dysfunction due to IRI primarily relies on clinical presentation and conventional biomarkers within the first 7–10 days after liver transplantation (LT). Additionally, liver metabolites have been used as biomarkers of IRI [61,62]. Notably, a decrease in total cholesterol levels within the first three days post-LT has been suggested as a predictive biomarker of three-month mortality [61,62]. Our observations revealed elevated levels of liver-type fatty acid-binding protein (L-FABP) throughout the perioperative period in grafts older than 65 years, with a significant difference in L-FABP levels noted on postoperative day (POD) 7 (Table 4). While some hepatic cell deterioration is expected following reperfusion in LT, our findings indicate a reduced regenerative capacity and a higher incidence of hepatocyte lysis in older grafts. These values did not align with our conventional biomarkers for liver function, nor did we observe a higher incidence of early postoperative complications or acute rejection in this subgroup, suggesting the presence of subclinical graft injury. An alternative explanation involves structural changes in older liver grafts, which are linked to hepatic artery fibrosis and atherosclerosis. These changes may result in subclinical hypoperfusion or hypo-oxygenation, particularly in the perivenous Zone 3 of the hepatocytes [63,64]. Analyzing donor FAPB-L levels at the time of multi-organ retrieval would provide valuable additional information. This analysis would allow for the assessment of dynamic changes in serum values and potentially determine the quality of liver grafts in marginal liver grafts. An extended follow-up study may be beneficial for elucidating the long-term effects of graft age on FABP levels. Such a study could potentially reveal accelerated deterioration and hepatocyte breakdown in older grafts, which may lead to increased rates of late postoperative complications or subclinical rejection of liver grafts. According to the literature, older donors have been associated with inferior outcomes and early mortality after LT, particularly in cases of donor-recipient age mismatch [10,11,12]. This may be attributed to the aging liver undergoing metabolic and immune alterations, resulting in increased production of pro-inflammatory cytokines, heightened liver lipid accumulation, and diminished regenerative capacity [11].

In pursuit of identifying serum biomarkers for liver graft damage, it is crucial to explore other research domains. MicroRNA (miRNA) profiling has shown that miR-483-3p and hsa-miR-885-5p can be used to diagnose acute cellular rejection [65]. miRNAs such as miR-122-5p, miR-194, miR-133a, and miR-148a-3p, extracted from bile, are notably elevated in cases of acute cellular rejection, thus serving as early prognostic indicators [66]. Post-liver transplantation cytokine levels, including IL-10, IL-17, and CXCL-10, can be used to predict liver graft rejection. Furthermore, the expression of exosome-derived galectin-9 is increased in patients undergoing acute liver rejection [67]. Additionally, microbiota analysis has identified specific changes in the microbial diversity of patients with acute liver rejection [68]. However, all these markers were studied in the context of acute rejection, without considering their implications during the early postoperative phase. Owing to the complexity of liver transplantation procedures and treatments, various scoring systems have been assessed as predictive factors for liver transplantation outcomes. The most widely used systems are the Model for End-Stage Liver Disease (MELD) and Albumin–Bilirubin Ratio (ALBI) [69,70,71,72]. Studies have linked higher MELD scores to more bile leaks and significantly longer ICU and hospital stays [73]. We did not find a significant correlation between preoperative ALBI or MELD scores, warm or cold ischemia time, and postoperative L-FABP levels.

Our study had several limitations. This was a single-center study with a limited sample size. Although we aimed to enroll a larger cohort within the study’s timeframe, the number of liver transplants performed depended on unpredictable factors such as the availability of suitable donors, which cannot be anticipated in advance. Another key factor contributing to the low number of cases is the high cost of FABP reagents used for blood sample evaluation. For future research, extending the data collection period may be beneficial, although this could pose challenges due to the prolonged storage of certain samples. Another limitation is the availability of donor FABP-L samples, which are essential for establishing the baseline status of liver grafts. Blood samples for FABP levels were collected on specific postoperative days (PODs); however, the time elapsed since reperfusion varied among patients, potentially leading to discrepancies in peak FABP values. Samples from all patients were analyzed within the same period, implying that some samples were stored for extended periods, which may have influenced the results. A viable approach to increasing the number of participants in the study would be to implement a multicenter research design. Given the limited sample size, it was not feasible to accurately determine a cutoff value for serum FABP levels that would indicate postoperative complications. Our data cannot determine whether the FABP-L marker is more suitable as an indicator of acute liver graft injury or if it is better utilized as a marker of subclinical liver graft injury at the cellular level, such as in cases of rejection or recurrence of autoimmune disorders. Regarding data analysis, values below the limit of detection (LOD) were substituted with LOD/2, consistent with standard practices for analyzing left-censored biomarker data in clinical and epidemiological research. This approach facilitates the retention of low, yet meaningful measurements while minimizing the bias introduced by listwise deletion. This substitution method has been supported by several studies. Ganser and Hewett (2010) demonstrated that the use of LOD/2 is a widely accepted and practical method for analyzing left-censored data, particularly when the proportion of values below the LOD is low [74]. Wood et al. (2011) acknowledged that although simple substitution can introduce bias, it remains suitable for preliminary analyses or when the data are otherwise robust [75]. Furthermore, in a large-scale biomarker study on liver decompensation and ACLF by Juanola et al. (2021), urinary L-FABP levels were analyzed as continuous variables, suggesting a similar approach to handling low values [29]. Despite its utility, this method has several limitations. It assumes a uniform distribution of values below the LOD and may underestimate the variability or bias of mean values if a substantial proportion of the data fall below the detection limit. Although the initial findings are promising, a larger sample size is required to assess L-FABP as a marker of liver graft injury at the cellular level in future studies.

## 5. Conclusions

Our findings indicate that L-FABP holds promise as a more sensitive biomarker for the early detection of subclinical hepatic graft cellular injury, which is not identified using conventional biomarkers for liver function. Nevertheless, larger cohort studies and long-term follow-up are necessary to evaluate whether postoperative assessment of L-FABP offers any clinical benefits.

## Figures and Tables

**Table 1:** Baseline patient characteristics and intraoperative details of liver transplant recipients; SD—standard deviation.

Patient Demographics	N = 29
Age, years—mean (SD)	55.21 (SD = 11.88)
Gender—n (%)	
Male	18 (62.1%)
Female	11 (37.9%)
Comorbidities—n (%)	
Pulmonary	5 (17.2%)
Cardiovascular	8 (27.6%)
Renal	8 (27.6%)
Diabetes mellitus	7 (24.1%)
Other	10 (34.5%)
Indication for transplantation—n (%)	
Alcohol-related liver disease	11 (37.9%)
Liver malignancy in cirrhotic liver	6 (20.7%)
Autoimmune	6 (20.7%)
Genetic/metabolic	3 (10.3)
Cryptogenic	2 (6.9%)
Other	5 (17.2%)
Intraoperative details	
Biliary anastomosis type—n (%)	
Ducto-ductal anastomosis	24 (82.8%)
Hepatico-jejunostomy	5 (17.2%)
Portocaval shunt—n (%)	14 (48.3%)
Warm ischemia, minutes—mean (SD)	44.2 (SD = 14.5)
Cold ischemia, minutes—mean (SD)	487.1 (SD = 96.4)

**Table 2:** Association between preoperative FABP-L levels and recipient age. MELD—model for end-stage liver disease, FABP-L—liver isomer fatty acid binding protein; Sig. = Significance (*p*-value).

Variable		FABP-L Before Surgery	MELD Score
FABP-L before surgery	Spearman’s rho	1.000	−0.344
	Sig.	/	0.085
MELD score	Spearman’s rho	−0.344	1.000
	Sig.	0.085	/

**Table 3:** Correlation between preoperative FABP-L levels and the ALBI score. ALBI—albumin bilirubin score; FABP-L—liver isomer fatty acid binding protein; Sig. = Significance (*p*-value).

Variable		FABP-L Before Surgery	ALBI Score
FABP-L before surgery	Spearman’s rho	1.000	−0.397
	Sig.	/	0.045
ALBI score	Spearman’s rho	−0.397	1.000
	Sig.	0.045	/

**Table 4:** FABP-L levels post-surgery between grafts from donors younger than 65 and those aged 65. FABP-L—liver isomer fatty acid binding protein, POD—postoperative day; SD = Standard Deviation; U = Mann–Whitney U statistic; r = Effect size; Sig. = Significance (*p*-value).

Variable		n	Mean	Median	SD	U	Z-Value	r	Sig.
FABP-L POD 1	Under 65	9	8	37.2	126.1	62.0	−0.122	0.03	0.928
	65 and older	16	16	39.2	34.3				
FABP-L POD 3	Under 65	10	10	18.4	19.8	43.0	−1.950	0.46	0.053
	65 and older	16	16	38.8	47.5				
FABP-L POD 5	Under 65	9	9	29.4	35.9	49.0	−0.882	0.22	0.403
	65 and older	15	14	41.4	99.6				
FABP-L POD 7	Under 65	9	9	38.6	29.2	32.0	−2.117	0.52	0.035
	65 and older	16	15	80.5	67.9				
FABP-L POD 14	Under 65	10	10	135.9	130.8	65.0	−0.791	0.18	0.452
	65 and older	15	16	102.3	112.4				

**Table 5:** Association between intraoperative blood transfusion volume and FABP-L levels. FABP-L—liver isomer fatty acid binding protein, POD—postoperative day; ml—milliliters; Sig. = Significance (*p*-value).

Variable		Blood Transfusion During Surgery (mL)
FABP-L POD 1	Spearman’s rho	−0.316
	Sig.	0.132
FABP-L POD 3	Spearman’s rho	−0.362
	Sig.	0.069
FABP-L POD 5	Spearman’s rho	−0.677
	Sig.	<0.001
FABP-L POD 7	Spearman’s rho	−0.455
	Sig.	0.025
FABP-L POD 14	Spearman’s rho	−0.256
	Sig.	0.206

**Table 6:** Summary of key findings. POD—postoperative day.

Subgroup	L-FABP Trends
Graft rejection	No statistical differences observed on PODs 1,3,5,7,14
Vascular complications	No statistical differences observed on PODs 1,3,5,7,14
Biliary complications	No statistical differences observed on PODs 1,3,5,7,14
Postoperative hemorrhage	Statistically significantly lower values for L-FABP on PODs 5 and 7
Grafts > 65 years	Statistically significantly higher values for L-FABP on POD 7

## Data Availability

Data are available upon request.
